# The impact of musical training in symbolic and non-symbolic audiovisual judgements of magnitude

**DOI:** 10.1371/journal.pone.0266165

**Published:** 2022-05-05

**Authors:** Nikos Chalas, Alexandros Karagiorgis, Panagiotis Bamidis, Evangelos Paraskevopoulos

**Affiliations:** 1 Institute for Biomagnetism and Biosignal analysis, University of Münster, Münster, Germany; 2 School of Medicine, Faculty of Health Sciences, Aristotle University of Thessaloniki, Thessaloniki, Greece; 3 Department of Psychology, University of Cyprus, Nicosia, Cyprus; University of Western Ontario, CANADA

## Abstract

Quantity estimation can be represented in either an analog or symbolic manner and recent evidence now suggests that analog and symbolic representation of quantities interact. Nonetheless, those two representational forms of quantities may be enhanced by convergent multisensory information. Here, we elucidate those interactions using high-density electroencephalography (EEG) and an audiovisual oddball paradigm. Participants were presented simultaneous audiovisual tokens in which the co-varying pitch of tones was combined with the embedded cardinality of dot patterns. Incongruencies were elicited independently from symbolic and non-symbolic modality within the audio-visual percept, violating the newly acquired rule that “the higher the pitch of the tone, the larger the cardinality of the figure.” The effect of neural plasticity in symbolic and non-symbolic numerical representations of quantities was investigated through a cross-sectional design, comparing musicians to musically naïve controls. Individual’s cortical activity was reconstructed and statistically modeled for a predefined time-window of the evoked response (130–170 ms). To summarize, we show that symbolic and non-symbolic processing of magnitudes is re-organized in cortical space, with professional musicians showing altered activity in motor and temporal areas. Thus, we argue that the symbolic representation of quantities is altered through musical training.

## Introduction

Organisms have biologically-endowed abilities for using their senses synergistically, allowing them to act complementary to each other [1]. Recent studies reveal that multisensory phenomena might originate on the basis of automated integration of convergent stimulus characteristics in time or space [2–4], on the statistics of cross-modal regularities [5–7], or on explicitly learned rules binding otherwise unrelated unisensory stimuli [8, 9].

On the other hand, humans—and other animals—acquire the ability to represent discrete sets of elements [10, 11]. Particularly, we estimate precisely, without counting, a small set of items [12]. The above-mentioned mechanism is present across different human societies [13] as it has been evolutionary conserved within a wide variety of other species–from bees to non-human primates—highlighting its importance for survival and its crucial role for social behavior [14–16]. There is a broad agreement that representations of numerosity are analog and multi-sensory: embedded within a sequences of sounds, in the cardinality of figures or in a series of actions [17]. On this basis, quantitative judgments for time or space of non-symbolic nature have been documented [12, 18], proposing a domain-general, or an amodal representation of analog quantities, underlying the computations of quantities, space, and time [19].

Relevant neuroimaging studies suggest an active role of the parietal cortex in this representational mechanism [20–22]. The traditional view on this cognitive mechanism presumes that these estimations are supported and expanded by the use of symbolic references representing quantities (e.g. numbers). A product of cultural acquisition, symbolic reference emerged late in human evolution [23] and established a powerful mental tool for representing any kind of associations between a symbol and its meaning [24]. However, recent empirical evidence supports the notion that distinct brain areas underpin the processing of notation-dependent (therefore symbol-dependent) and independent quantities [25], pointing towards the existence of two quantity-processing mechanisms: one that processes symbol-dependent quantities and another that processes analog ones [26]. Nevertheless, it is still unclear whether any of those representational mechanisms are subject to change due to the long-term effects of cultural acquisition as provided by education and training, or whether their development is phylogenetically predetermined.

Our brains are effectively interacting with the environment, which shapes its properties and determines both its structure and function. In the neural level this could be achieved by the strengthening of synapses [27, 28], although the exact mechanisms underlying this plasticity are still called into question. Interestingly, musical training is one such model to study neural plasticity [29, 30], since playing a musical instrument involves the partition of several—perfectly tuned—sensory systems. Musical training constitutes a powerful stimulator of neuroplasticity [31, 32] and enculturation [33]. As musical expertise requires long-term training, several studies use a cross-sectional approach, comparing musicians to non-musicians, providing indices of training-induced plasticity [29]. Numerous studies have been reported for auditory, multisensory, or motor tasks [for review: [29]] with stimuli confined to the nature of musical action. Neuroplastic changes in tasks with stimuli outside the borders of music have also been reported [34–38]. Those studies indicate changes in domain-general cognitive mechanisms that are related to musical training [39]. Behaviorally, it has been shown that musicians outperform musically naïve individuals in magnitude estimation at temporal, spatial and numerical discrimination tasks [40]. To this end, a study by Proverbio et al. (2013) used EEG and an ERP design to find an increased activity of the fusiform and inferior occipital gyrus during word reading comparing musicians to non-musicians, indicating cortical reorganization of symbolic representations in the language domain [41].

A series of experiments have extensively investigated the neurophysiological underpinnings of audiovisual integration via event-related-potentials in linguistic [42, 43] and musical stimuli [44, 45] with Magneto- or Electro-encephalography (M/EEG) using a common neuropsychological index, called the Mismatch-Negativity paradigm [46]. During a MMN paradigm, infrequent incongruencies (~20% of stimuli) are interleaved within a string of congruent ones (~80% of stimuli), yielding an error-related activity in the brain (approx. 150ms), resulting from the top-down comparison of an internal prediction [47] with the bottom-up sensory evidence. Thus, MMN components provide fertile ground to contrast experimental conditions representing cognitive processes. Traditionally, MMNs were studied within the auditory domain and are triggered by “surprising” frequencies, durations or amplitudes of sound stimuli. Similar mechanisms have been reported for the processing of visual [48] and somatosensory [49] stimulation, indicating a common cross-modal mechanism for the detection of deviants across previously-learned regularities. Paraskevopoulos et al. (2014) used a multi-feature MMN paradigm in which pitches were combined with symbolic numbers with an explicitly acquired rule “the higher the pitch of the tone, the larger the number of the figure” [50]. They showed that musical expertise is related to increased activity in the prefrontal cortex during multisensory judgments of magnitude within 130–170 ms of the ERP response. Nonetheless, the design of the abovementioned study did not allow the differentiation between the effects of symbolic and non-symbolic representation of quantity processing. Here, we adapt this paradigm and we include a condition in which cardinality is coded in a non-symbolic manner to elucidate the effect of symbolic and non-symbolic numerical processing.

The present study aims to disentangle *symbolic quantities* from *non-symbolic quantity* processing and the effect that musical training has on this interaction by comparing neural responses of musicians to non-musicians. To this aim, Electroencephalographic (EEG) data were used to estimate cortical responses in an adapted multisensory MMN paradigm [44, 50]. In this paradigm, a stream of congruent audiovisual quantities were interleaved with “surprising” violations equivalent to traditional MMN paradigms. Incongruencies were elicited independently within either audiovisual symbolic or non-symbolic representations. Audiovisual congruency was defined according to the explicit rule: “the higher the pitch of the tone, the larger the cardinality of the figure” and audiovisual stimuli that didn’t follow this rule (symbolic or non-symbolic) were considered as incongruent. The effect of long-term plasticity due to musical training in the corresponding incongruency response generators was evaluated via a comparison of musically trained individuals to musically naïve controls in the predefined window of 130–170 ms after stimulus presentation. Our main hypothesis was that musical training enhances the ability to represent numbers in a two-fold manner, by supporting both the analog and notation-independent quantities along with symbolic and notation-dependent representations. Previous research on brain plasticity associated with musical training has revealed that perceptual changes attributed to long-term musical training are domain general and are related to auditory and motor systems [29, 32, 51]. Thus, in our multi-feature oddball paradigm we were expecting activation differences in cortical generators of the embedded incongruencies in temporal and motor areas between musicians and non-musicians, consistent with prior research in a similar paradigm [50].

## Materials & methods

### Participants

The sample of the study was 30 individuals: 15 musicians and 15 non-musicians. Musicians (mean age = 26.44 years, SD = 3.02 years, 7 males) were professional musicians or students of Music Studies (mean musical training = 15.47; *SD* = 3.72). Non-Musicians (mean age = 23.34, SD = 4.45, 8 males) had not received musical lessons apart from compulsory school lessons. Sample size was calculated via G-Power on the basis of a power analysis of the behavioral data measured in Paraskevopoulos et al., (2014). This analysis revealed an effect size of 0.98; targeting a one-tailed analysis. The sample size analysis revealed that the desired size for each group is 14 subjects, to reach a power of 0.8 and significance level of a = 0.05. All participants had normal hearing and normal -or corrected to normal- vision. The study was approved by the ethics committee of the Medical Faculty of University of Thessaloniki, whereas the study is in accordance with the Declaration of Helsinki (1964). Before the EEG session, participants signed written consent.

### Stimuli

**Fig 1:**
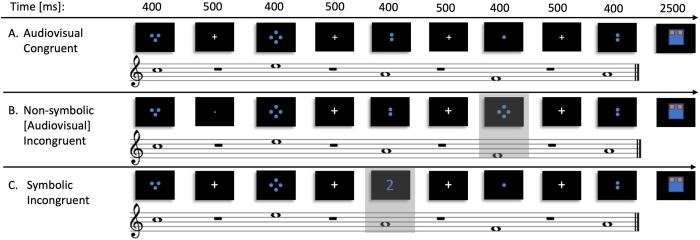
Illustration of the design. Example of congruent and incongruent trials. A: Audiovisual congruent trial was based on the explicit rule “the higher the pitch of the tone, the larger the cardinality of the figure” B: Audiovisual incongruent trial contained an incongruent stimulus with respect to the learned rule. C: Symbolic incongruent trial contained visual stimulus in which cardinality was presented through symbol (Arabic digit).

Congruent and incongruent stimuli were prepared by combining 4 sinusoidal tones (F5, 698.46 Hz; A5, 880.46 Hz; C6, 1046.50 Hz; and E6, 1318.51 Hz) to five-tone melodies. Figures illustrating disc-patterns (1 dot up to 5 dots, in blue color) were presented simultaneously with each sinusoidal tone. Tones had a duration of 400ms, including 10ms rise and decay time and a sampling rate of 44.100 Hz. The Inter Stimulus Interval (ISI) was set to 500ms and the total duration of each melody was 4 sec. During ISI a fixation cross was presented. Finally, 8 melodies were prepared for each video category. For each tone, the figure was presented in the exact time and for the same duration with the tone (**Fig 1**).

Congruency of stimuli (A) followed the instructed rule: “the higher the pitch of the tone, the larger the cardinality of the figure”. Incongruent categories included violation in the (B) audiovisual modality: the co-varying pitch of the tone and cardinality of the presented figure did not match according to the abovementioned congruent rule and (C) symbolic modality: the figure encoded magnitude information through symbolic quantities (Arabic-digit instead of analog patterns). For sanity checking of our study with MEG dataset [44, 50] the paradigm included also violations of the auditory modality: the presented tone was delivered with different timbre (piano instead of sinusoidal tone) and visual modality: the discs were either presented with different color (red instead of blue). Auditory and visual incongruencies were outside the scope of the current study and thus are not reported herein. Each melody has a ratio of 4:1 congruent to incongruent stimuli, for each modality independently (**Fig 1**).

### EEG recordings

Evoked Related Potentials were recorded with a Nihon-Kohden 128 channel EEG recording system and an active electrodes cap (actiCAP128, Brain Products), inside an electrically shielded room. Data were acquired continuously with a sampling rate of 500 Hz, with electrode impedance kept below 10 kΩ, while participants were seated comfortably in an upright position. Auditory stimulation was delivered via headphones at 60 dB above each participant’s hearing threshold, determined at the beginning of the EEG session. Visual stimuli were delivered through a flat-panel display located ~ 110 cm away from the subject, with a refresh rate of 60 Hz and a spatial resolution of 1280 x 768 pixels.

### Experimental design

A multi-feature oddball paradigm was generated by combining random videos from each condition, forming blocks. After each 4sec video, participants were asked to decide whether the previously presented video violated the instructed rule “the higher the pitch of the tone, the larger the cardinality of the figure”. In total, there were 3 blocks of 14.5 mins, with short breaks in between. Finally, the total amount of trials was 72 for each condition.

### Data analysis

#### Behavioral data analysis

For estimating the accuracy of behavioral responses we calculated the discriminability index d-prime [52] on the 2AFC task. The 2AFC task was placed after the presentation of each video (containing the five-tone melody). Participants had to decide whether there was -or not- a violation of the acquired rule “The higher the pitch of the tone, the larger the cardinality of the dots”, within the previously presented video. Thus, the responses of audiovisual symbolic and non-symbolic (in)congruencies were evaluated. Individual responses to each video taken from three blocks of EEG measurements were collected for the calculation of the d-prime. Data were analyzed with a 2×2 mixed model ANOVA with between-subjects factor group (musicians, non-musicians) and within-subject condition (non-symbolic, symbolic). The significance level was set to p < 0.05.

#### EEG data pre-processing

The Brain Electrical Source Analysis software (BESA Research, version 6; Megis Software) was used to process the EEG data. The data were visually inspected: bad channels were interpolated and artifacts due to eye-movement were corrected through an adaptive artifact correction [53]. Continuous data were separated into epochs of 800 ms, including 200 ms of a pre-stimulus interval, serving as a baseline. As stimulus, we considered one single sinusoidal tone (and the corresponding figure) within each presented five-tone melodies. Data were filtered offline with a Butterworth high pass forward filter of 1 Hz, a Butterworth low pass zero-phase filter of 30 Hz, and a notch filter at 50 Hz. Epochs were averaged separately, resulting in 3 conditions (1 congruent and 4 incongruent). An equal number of epochs (n = 72) was chosen for each experimental condition for ensuring equal signal-to-noise ratio across conditions. Similar analytic approach has been applied and verified in previous studies [54, 55].

#### Source activity estimation

Individual’s source activity was estimated for each condition using the low-resolution electromagnetic tomography (LORETA) [56]. LORETA provides computation of distributed currents across the whole-brain, while maintains the advantage of not needing an a-priori definition of activated sources. Based on previous research [44], an appropriate time-window of 130–170 ms across each condition was chosen for analysis. Current Density Reconstructions (CDRs) of the selected time-window were projected onto an MRI template based on the Montreal Neurological Institute (MNI). Head models were simulated realistically with a Finite Element Model (FEM), using an average of 50 individual MRIs.

Furthermore, the images were smoothed through an isotropic Gaussian kernel with 7mm full-width half-maximum as provided by BESA.

#### Statistical analysis of EEG data

Current Density Reconstructions (CDRs) were coregistered into the MNI space with Statistical Parametric Mapping 12 (SPM 12, https://www.fil.ion.ucl.ac.uk/spm) and statistically modeled using the Sandwich Estimator Toolbox [SWE; [57]] implementing a flexible factorial model -equivalent to 2x3 mixed model ANOVA with within-subject factor Condition [congruent, audiovisual incongruency, symbolic incongruency] and between-subject factor Group (musicians and non-musicians). We note that, in the behavioral level in which d-prime was calculated, within-subject conditions were 2 (non-symbolic and symbolic) as the congruent condition was used for the calculation of d-prime for both of the conditions. In the source analysis results, the congruent condition was also included in the model (and thus having 3 levels) as the difference between congruent and incongruencies (symbolic and non-symbolic) is a marker of the typical MMN response. We note that throughout the manuscript we refer to the statistical difference between congruent and each incongruent condition as “mismatch”. Finally, a Monte-Carlo approach was applied [58] by which simulated statistic images were compared under the null hypothesis and thus setting the maximum cluster size, above which p-values correspond to corrected p-values.

## Results

### Behavioral responses

**Fig 2:**
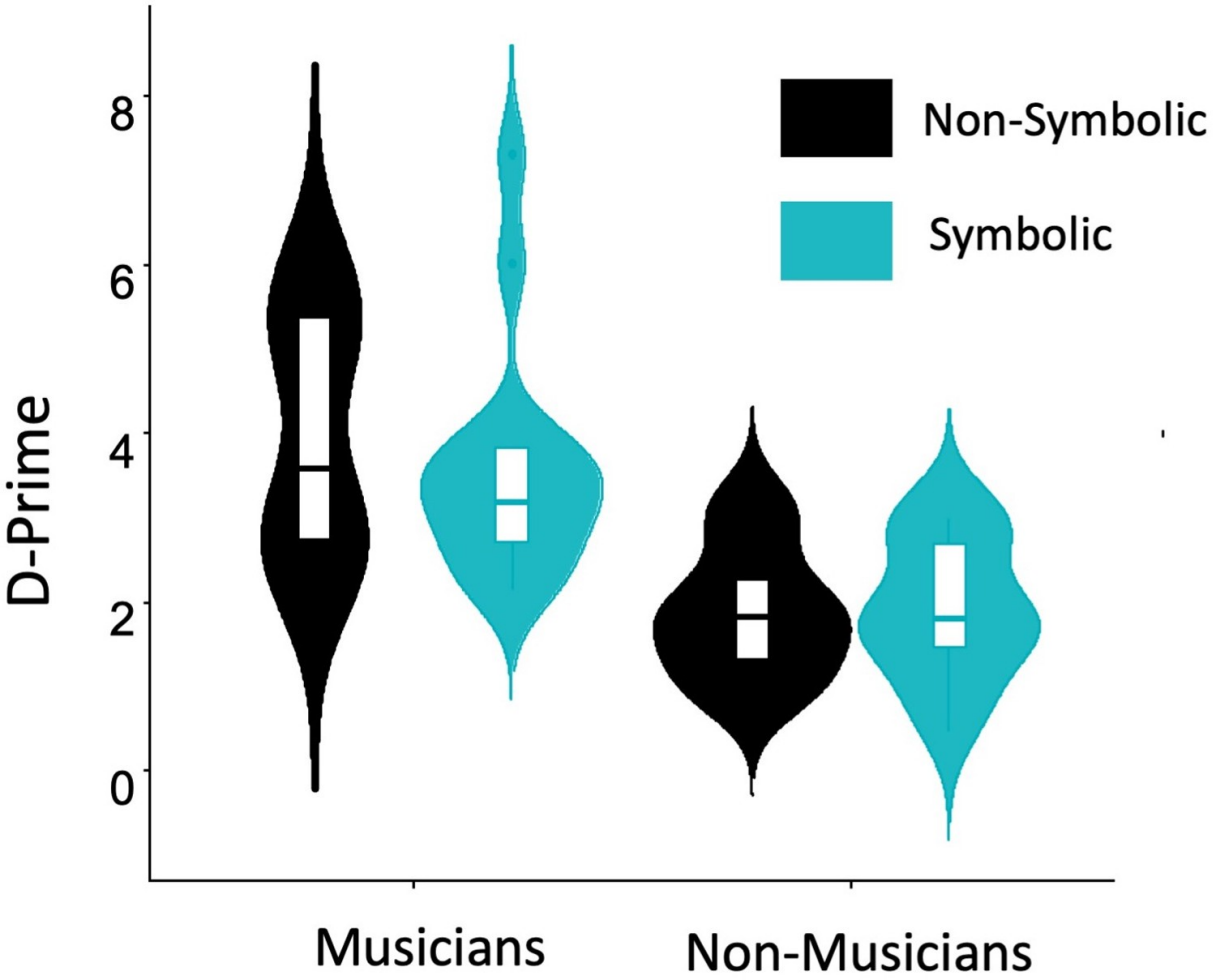
Behavioral responses. D-prime values for musicians and non-musician in non-symbolic [audiovisual] incongruencies (black) and symbolic incongruencies (blue).

For measuring participant’s ability to detect whether a presented video congruent or incongruent, we calculated the discriminability index, d-prime [52] for the audiovisual (non-symbolic) and symbolic modality. The statistical analysis of the behavioral responses revealed a significant main effect of the factor Group $\left[F(1,28)=27.99,\;p=0.000,\;\eta_{p}^{2}=0.5\right]$, indicating enhanced accuracy in musicians’ behavioral responses, irrespective of the experimental conditions. The main effect of factor Condition $\left[F(1,28)=.784,\;p=0.383,\;\eta_{p}^{2}=0.027\right]$ and the interaction of Group × Condition $\left[F(1,28)=1.03,\;p=0.319,\;\eta_{p}^{2}=0.035\right]$ didn’t reach significance, indicating that the experimental conditions (audiovisual symbolic and non-symbolic incongruencies) didn’t have an effect on the behavioral responses of participants. In Fig 2 we summarize the difference in d-prime for musicians and non-musicians.

### EEG source activity

**Fig 3:**
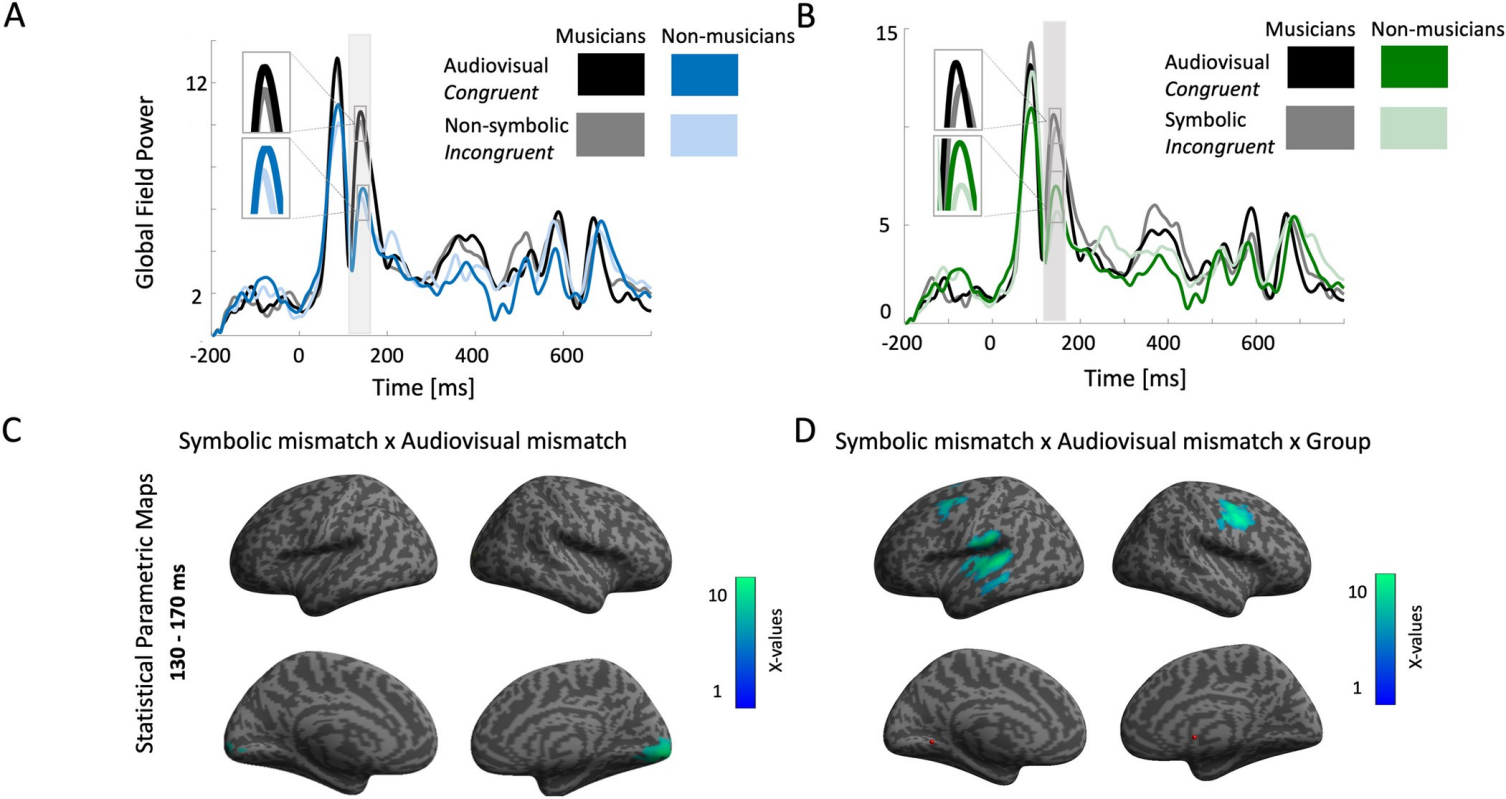
Audiovisual non-symbolic and symbolic interaction in musicians and non-musicians. A: Grand average of global field power for musicians and non-musicians during the presentation of audiovisual congruent and audiovisual (non-symbolic) incongruent stimuli. Shaded area depicts the predefined window of analysis (130–170 ms). B: Grand average of global field power for musicians and non-musicians during the presentation of audiovisual congruent and audiovisual symbolic incongruent stimuli. Shaded area depicts the predefined window of analysis (130–170 ms). C: Statistical Parametric Maps for the interaction of audiovisual non-symbolic and symbolic mismatch response generators. Threshold p<0.005, cluster corrected. D: Statistical Parametric Maps for the interaction of audiovisual non-symbolic mismatch × symbolic mismatch × group. Threshold p<0.05, cluster corrected.

Firstly, as we were interested in assessing the neural interaction of symbolic and analog processing of numerosity in audiovisual context we proceeded with the calculation of Audiovisual mismatch × Symbolic mismatch interaction. This statistical analysis yielded one significant cluster located at left occipital cortex [peak coordinates: x = 16, y = -96 z = -18; X(28) = 11.83; cluster size = 3485 voxels; p < 0.005 (corrected); **Fig 3, panel C**].

To evaluate the effect of musical training, we proceeded with the calculation of Audiovisual mismatch × Symbolic mismatch × Group interaction. This interaction yielded three statistically significant clusters, located at: a) left superior temporal gyrus [peak coordinates: x = -58, y = -26 z = 12; X(28) = 7.19; cluster size = 3648 voxels; p<0.05 (corrected)], b) left SMA [peak coordinates: x = 42, y = -6, z = 38; X(28) = 6.54; cluster size = 1846 voxels; p < 0.05 (corrected)] c) right SMA [peak coordinates: x = -36, y = 4, z = 56; X(28) = 5.61; cluster size = 1316 voxels; p < 0.05 (corrected); **Fig 3, panel D**].

**Fig 4:**
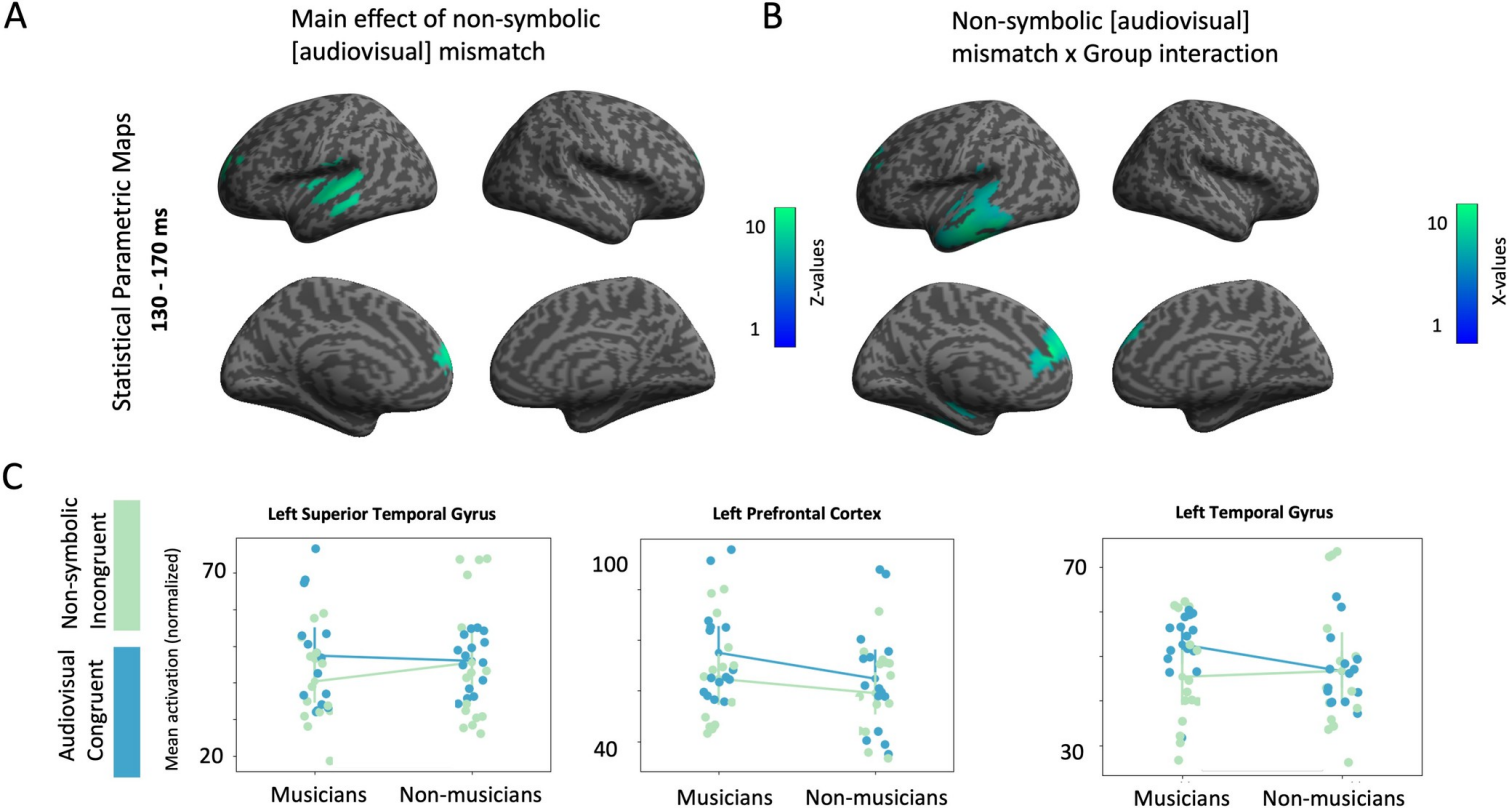
Audiovisual non-symbolic mismatch response generators in musicians and non-musicians. A: Statistical Parametric Maps for the main effect of audiovisual non-symbolic incongruency. Threshold p<0.005, cluster corrected. B: Statistical Parametric Maps for the interaction audiovisual non-symbolic mismatch × group. Threshold p<0.05, cluster corrected. C. Point plots depicting individual’s and mean activity for each statistically significant cluster of activation for the main effect of audiovisual non-symbolic mismatch and audiovisual non-symbolic mismatch × group interaction.

For unraveling the impact of non-symbolic audiovisual incongruency, we proceeded with the calculation of the main effect of audiovisual mismatch. Our results indicated two significant clusters of activation: a) one cluster located at left superior temporal gyrus [peak coordinates: x = -24, y = 62, z = 30; Z(28) = 3.10; cluster size = 2166 voxels; p < 0.005 (corrected); **Fig 4, panel A**] and b) one cluster located at left pre-frontal cortex [peak coordinates: x = -66, y = -16, z = 2; Z(28) = 3.42; cluster size = 2856 voxels; p < 0.005 (corrected)]. For those two clusters, non-musicians exhibited lower activation in the audiovisual congruent condition and higher in the non-symbolic audiovisual incongruent compared to musicians, and for the left pre-frontal cortex, non-musicians showed decreased activation for both non-symbolic audiovisual congruent and incongruent response. At the left superior temporal gyrus, musicians showed higher activity in the non-symbolic audiovisual congruent and lower activation in the non-symbolic audiovisual incongruent, when compared to non-musicians (**Fig 4, panel C)**. Furthermore, for assessing the impact of musical training in audiovisual incongruency response generators we calculated the Audiovisual incongruency × Group interaction. This statistical analysis yielded two significant clusters of activation: a) one cluster located at left temporal cortex [peak coordinates: x = -16, y = -44, z = 22; Z(28) = 2.53; cluster size = 3510 voxels; p < 0.005 (corrected)] and b) one cluster located at left pre-frontal cortex [peak coordinates: x = -56, y = -32, z = -34; Z(28) = 2.41; cluster size = 5840 voxels; p < 0.005 (corrected); **Fig 4, panel B**]. Similar to the left superior temporal gyrus pattern, at the left temporal gyrus musicians showed increased activation for the audiovisual congruent condition and decreased activation for the non-symbolic incongruent condition, when compared to non-musicians (**Fig 4, panel C**).

**Fig 5:**
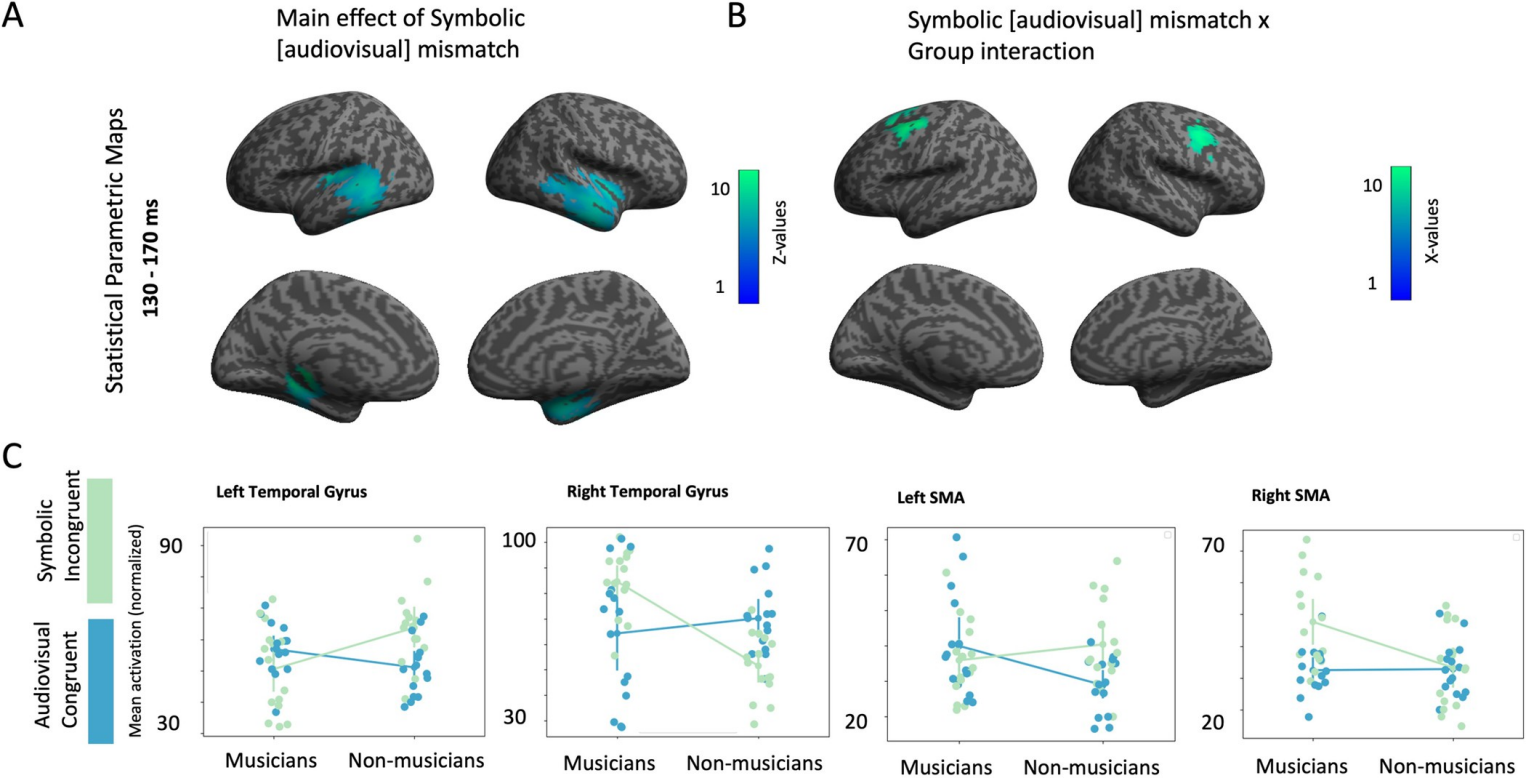
Audiovisual symbolic mismatch response generators in musicians and non-musicians. A: Statistical Parametric Maps for the main effect of audiovisual symbolic mismatch. Threshold p<0.005, cluster corrected. B: Statistical Parametric Maps for the interaction audiovisual symbolic mismatch × group. Threshold p<0.05, cluster corrected. C. Point plots depicting individual’s and mean activity for each statistically significant cluster of activation for the main effect of audiovisual symbolic mismatch and audiovisual symbolic mismatch × group interaction.

Furthermore, for assessing the impact of symbolic incongruency, we proceeded with the calculation of the main effect of symbolic incongruency. The statistical analysis yielded two significant clusters of activations: a) one located at left temporal cortex [peak coordinates: x = -16, y = -36, z = -4; Z(28) = 2.91; cluster size = 4914 voxels; p < 0.05 (corrected)] and b) one in right temporal cortex [peak coordinates: x = 34, y = -14, z = -12; Z(28) = 2.23; cluster size = 7017 voxels; p < 0.05 (corrected); **Fig 5, panel A**]. At the left temporal gyrus, musicians showed increased activation in the audiovisual congruent and decreased activation in the symbolic incongruent condition, when compared to non-musicians (**Fig 5, panel C**). Strikingly, at the right temporal gyrus we observed the opposite pattern: musician’s showed increased activation at the symbolic incongruent condition and decreased at the audiovisual congruent condition, when compared to non-musicians. Additionally, for evaluating the effect of musical training in the symbolic incongruency response, we calculated the Symbolic mismatch × Group interaction. This statistical analysis yielded two significant cluster of activations: a) one located at left SMA [peak coordinates: x = -36, y = 0, z = 52; Z(28) = 2.19; cluster size = 2200 voxels; p < 0.05 (corrected)] and b) one located at right SMA [peak coordinates: x = 32, y = 4, z = 38; Z(28) = 2.09; cluster size = 1828 voxels; p < 0.05 (corrected); **Fig 5, panel B**]. Similar to the observation for the left and right temporal gyrus, at the left SMA musicians showed increased activation for the audiovisual congruent condition and decreased activation for the non-symbolic incongruent condition when compared to non-musicians, whereas the opposite effect was observed for the right SMA (**Fig 5, panel C**).

As our results may have been driven by group characteristics that are not directly tested in this study, we tested whether the activation strength of each cluster identified via the interaction of Symbolic mismatch x Audiovisual mismatch x Training in the sub-group of musicians can be predicted by the amount of musical training, namely the number of years since training began. The clusters included in the analysis were the left Auditory Cortex, the right SMA and the Occipital Cortex. The result of this analysis identified that the activation strength of the left Auditory Cortex when processing symbolic incongruencies can be significantly predicted by the amount of training [*b* = .674, *t*(9) = 2.58, *p* = .033]. The rest of the clusters included in the analysis did not yield a significant effect. This serves as evidence arguing for the grounding of this difference in musical training instead of innate characteristics of the groups.

In Table 1 we summarize the individual’s source activation values (mean ± SD) from each significant cluster yielded from the analysis.

**Table 1:** Activity of each cluster for each contrast evaluated.

Anatomical Region	Group	Condition	Activation (mean ± SD)
Left STG	Musicians	Audiovisual Standard	47.41 ± 13.78
		Audiovisual Deviant	40.34 ± 11.21
	Non-musicians	Audiovisual Standard	45.95 ± 7.08
		Audiovisual Deviant	45.51 ± 17.93
Left PFC	Musicians	Audiovisual Standard	74.93 ± 18.49
		Audiovisual Deviant	64.68 ± 19.08
	Non-musicians	Audiovisual Standard	64.73 ± 21.24
		Audiovisual Deviant	59.02 ± 16.04
Left Temporal Gyrus	Musicians	Audiovisual Standard	52.64 ± 7.13
		Audiovisual Deviant	45.39 ± 11.72
	Non-musicians	Audiovisual Standard	46.64 ± 7.51
		Audiovisual Deviant	46.71 ± 14.91
Left Temporal Cortex	Musicians	Audiovisual Standard	56.89 ± 8.24
		Symbolic Deviant	50.5 ± 13.4
	Non-musicians	Audiovisual Standard	51.07 ± 8.82
		Symbolic Deviant	63.72 ± 11.84
Right Temporal Cortex	Musicians	Audiovisual Standard	64.25 ± 25.44
		Symbolic Deviant	84.57 ± 12.23
	Non-musicians	Audiovisual Standard	70.19 ± 13.78
		Symbolic Deviant	51.51 ± 12.11
Left SMA	Musicians	Audiovisual Standard	39.86 ± 14.21
		Symbolic Deviant	35.81 ± 10.50
	Non-musicians	Audiovisual Standard	28.77 ± 7.52
		Symbolic Deviant	40.29 ± 11.98
Right SMA	Musicians	Audiovisual Standard	32.45 ± 14.21
		Symbolic Deviant	47.62 ± 13.76
	Non-musicians	Audiovisual Standard	32.81 ± 8.15
		Symbolic Deviant	33.25 ± 12.19

## Discussion

The present study used Electroencephalography (EEG) to investigate the cortical responses of audiovisual incongruencies, in which non-symbolic and symbolic magnitude information was combined with pitch-elevation in a multisensory mismatch paradigm. Effects of experience-driven neuroplasticity were monitored cross-sectionally, through the comparison of musically naïve individuals with professional musicians. Results indicated that the interaction of neural responses for symbolic and non-symbolic processing of magnitude originated in occipital sources. Interestingly, the interaction of musical training in this effect suggests that the neural activation for symbolic and non-symbolic incongruency differs between musicians and non-musicians for left superior temporal gyrus and bilateral motor areas. It is essential to note that our study followed a cross-sectional design and thus following the nature versus nurture debate our interpretation focuses on the effects of musical expertise, rather than the effect of training [59]. To summarize, our results indicate a re-organization of the neural processing of symbolic and non-symbolic magnitude information, possibly attributed to musical expertise.

On the behavioral level, both musicians and non-musicians were able to discriminate cross-modal correspondences when cardinality was coded either in an analog or symbolic manner. As expected, musicians were significantly better at detecting cross-modal incongruencies, confirming previous studies with a similar design [44, 50]. Nevertheless, there were no—within-group—significant differences in the detection of violation across symbolic and non-symbolic conditions, indicating that on a perceptual level, neither dots nor Arabic digits interfere with cross-modal decisions. To our knowledge, this is the first study investigating variations in multisensory judgments of magnitude that are attributable to symbolic and non-symbolic stimuli. Although considering uni-sensory environment and quantities above the subitizing range, our results confirmed previous findings in adults [60] and in children [61], even though opposite results exist as well [62]. Hence, our behavioral results regarding symbolic and non-symbolic quantity processing should be taken into consideration cautiously.

Regarding non-symbolic and symbolic audiovisual incongruency response generators, the symbolic mismatch × audiovisual mismatch interaction yielded one significant cluster of activation covering occipital gyrus. This is consistent with previous studies using MEG dipole modeling, which have reported visual MMN located at the MOG, peaking around ~150 ms after infrequent stimulus onset [63, 64]. This verifies its role for the detection of visual changes, even though the specific mechanism remains elusive [65, 66]. Results of the present study provide further evidence that the neural generator corresponding to the interaction of the symbolic and audiovisual (non-symbolic) mismatch covered occipital resources, indicating that the interaction of those incongruencies is perceived as a visual mismatch, as evident in a previous study [50]. The source analysis results of symbolic mismatch × audiovisual mismatch × group interaction revealed the effect of long-term musical expertise in the process. Specifically, the source analysis of the symbolic mismatch × audiovisual mismatch × group interaction was located in a cluster covering the left superior temporal gyrus (STG). Thus, along with our behavioral evidence, the symbolic mismatch × audiovisual mismatch × group interaction illustrates the enhanced ability of professional musicians at detecting uni- and cross-modal irregularities [39, 67], explained partly by neuroplastic changes of extensive multisensory training within the auditory cortex [51]. Two clusters, covering motor areas bilaterally, were also evident, confirming the reorganized audio-visuomotor processing in professional musicians during the perception of irregularities [68]. The right precentral gyrus has been previously found with fMRI to overlap during size and luminance comparison tasks of symbolic numbers [69], demonstrating the contribution of motor-association regions for domain-general magnitude comparisons.

Audiovisual non-symbolic and symbolic mismatch responses dissociated those processes from their corresponding interaction. The source analysis results for the audiovisual non-symbolic mismatch revealed that generators were located in the left superior temporal gyrus (STG) and middle frontal gyrus. Regarding frontal regions, middle frontal gyrus (MFG) is considered to support working memory and goal-oriented attention [70, 71], consistent with our experimental design, as participants were challenged to decide upon cross-modal correspondences after the completion of each block. The activation of the left superior temporal gyrus (STG) in audiovisual non-symbolic mismatch responses indicates that participants—regardless of musicianship—allocated temporal cortical sources for the cross-modal perception of incongruency. In a previous study with a similar paradigm [44], activation of the STG was found during the identification of auditory incongruencies. In the aforementioned study audiovisual regularities were acquired on the basis of pitch (auditory) and elevation (spatial). Nonetheless, in both studies [current and [44]], (in)congruency depended on the relation of each stimulus to the previous one, which is used as perceptual prior. Associations relying on absolute characteristics of each stimulus evoke innate [72] processing strategies that rely on cross-modal correspondences [73], but cannot be employed when the relative position designates the association. In the latter case, explicitly-learned rules have to be employed to lead congruency identification. Here, cross-modal correspondences incorporated information of magnitude in the visual modality. Hence, we argue that analog audiovisual incongruency responses constitute a domain-general mechanism, in which the corresponding cortical resources exhibit stimulus specificity within each modality.

The results of the source analysis of the interaction between the audiovisual non-symbolic incongruency response and the groups of musicians and non-musicians revealed increased activation in temporal and frontal areas, which might be attributed to the long-term plasticity effects of musical training. Specifically, musicians’ audiovisual mismatch response generators were located at a broad cluster in temporal region corroborating the notion that professional musicians prioritize their cortical resources on the auditory modality [74, 75]. Likewise, activation in the left superior frontal gyrus (SFG) indicates the audio-visuomotor processing of musical notation that musicians have acquired during their long-term training [76]. A relatively recent meta-analysis of Sokolowski et al. (2017) has reported a right-lateralized frontoparietal network in which superior parietal lobule, inferior parietal lobule, and superior frontal gyrus (SFG) support the processing of non-symbolic magnitude information [[77], see also: [71]]. Thus, our results suggest that SFG may be related to the analog and format-independent processing of quantities, along with high-association goal-oriented functions [78].

The main effect of symbolic mismatch was unfolded broadly in the bilateral temporal cortex, extending to inferior temporal gyrus (ITG). Interestingly, our results may be in part interpreted within the framework of the Triple Code model [10, 79] whereby numerical representations incorporate symbolic, verbal, and analog quantities. Accordingly, our results indicate that symbolic representation of quantities may be generated through bilateral activation of the temporal cortex, within a unitary audio-visual representation [80]. Abboud et al. (2015), through visual-to-music substitution paradigm and fMRI recordings, found distinct activation in right ITG in sighted and blind subjects, suggesting that its activity is independent of sensory income or experience [81]. Recently, studies trying to unravel the role of the ITG in numerical cognition applied intracranial electroencephalography (iEEG) and highlighted its role during perception of visual numerals [82, 83] as well during calculations [84]. ITG has also been found to be functionally connected with the lateral parietal cortex [LPC; [85]], a central hub for domain-specific representation of quantities [86–88], especially when noted in an analog manner [89] along with domain-general magnitude estimations of non-symbolic nature [21].

Activation of bilateral SMA was revealed at the group × condition interaction for the symbolic modality, indicating increased activity in the region, due to neuroplastic effects of musical expertise. This activation matches with the two motor-associated clusters revealed in the symbolic mismatch × audiovisual mismatch × group interaction and thus elucidates its origin. Τhe significantly-higher activation of motor areas delineates that the cortical re-organization of professional musicians affects the processing of numerical symbols [29, 76, 90]. Interestingly, when we look at activation patterns at the left and right SMA we observe an opposite effect between musicians and non-musicians. Specifically, while musicians show increased activation in the symbolic incongruency at the right SMA, they show decreased activation at the left SMA for the same condition. Similar pattern is also observed at the left temporal gyrus: musician’s show increased activation at the right temporal gyrus and decreased activation at the left temporal gyrus for the symbolic incongruency, when compared to non-musicians. Taken together, we observe a right-hemispheric dominance for the identification of symbolic [audiovisual] incongruencies for the group of musicians when compared to non-musicians, a finding consistent with previous results obtained from a longitudinal study [91].

Previously, it has been demonstrated that musical training enhances the association between pitch and spatial representation behaviorally [92] and neurophysiologically [44] Thus, our results may partly be driven by the enhanced audiovisual integration that musicians exhibit, evidenced even in primary auditory areas [51]. This enhancement may be transferred to the symbolic and non-symbolic processing of numbers. On the other hand–and more specific to visual modality–, musical training has been related to enhanced ability in processing of local features in a visual scene [93] which can explain the difference in processing non-symbolic and symbolic representation. We speculate that both these factors may ground the neuroplastic effects on symbolic and non-symbolic incongruence processing. Distinguishing which factor drives the corresponding effect remains outside of the scope of the current study, and requires further research

## Conclusion

We have found that cortical activity supporting symbolic quantities is subject to changes due to enculturation factors such as musical expertise. Ιncreased activation within the group of musicians, when compared to musically naïve controls, was evident in temporal and motor areas. In a theoretical framework, our interpretation collaborates the notion that symbolic reference–as a tool for the representation of exact quantities- has emerged via ‘the interaction of biology and culture [94, 95]. That is to say, interaction between the culturally-transmitted symbolic reference quantities and biologically-evolved quantity estimations seem to have acted synergistically during the course of evolution. Here, we provide evidence of this interaction, showing that culturally transmitted traits (i.e musical expertise) alters the cortical representation of non-symbolic and symbolic reference of quantities. Hence, we argue that the so-called ‘number faculty’ could not have been merely evolved through processes of natural selection but stands upon biologically grounded and phylogenetically conserved neuronal networks underpinning quantity representation [96].

## Abbreviations

EEG: electroencephalography
MMN: Mismatch Negativity
ISI: inter-stimulus interval
2AFC: two-alternative forced choice
CDR: current density reconstruction
SMA: supplementary motor area
MOG: middle occipital gyrus
STG: superior temporal gyrus
MFG: middle frontal gyrus
SFG: superior frontal gyrus
ITG: inferior temporal gyrus
